# Trade-off between photosymbiosis and innate immunity influences cnidarian’s response to pathogenic bacteria

**DOI:** 10.1098/rspb.2024.0428

**Published:** 2024-10-02

**Authors:** Madison A. Emery, Kelsey M. Beavers, Emily W. Van Buren, Renee Batiste, Bradford Dimos, Mark W. Pellegrino, Laura D. Mydlarz

**Affiliations:** ^1^ Department of Biology, University of Texas at Arlington, Arlington, TX 76019, USA; ^2^ Department of Integrative Biology, Michigan State University, East Lansing, MI 48824, USA; ^3^ Texas Advanced Computing Center, University of Texas at Austin, Austin, TX 78758, USA; ^4^ Department of Animal Sciences, Washington State University, Pullman, WA 99163, USA

**Keywords:** symbiosis, innate immunity, trade-offs, mutualism, cnidarian, jellyfish

## Abstract

Mutualistic relationships with photosynthetic organisms are common in cnidarians, which form an intracellular symbiosis with dinoflagellates in the family Symbiodiniaceae. The establishment and maintenance of these symbionts are associated with the suppression of key host immune factors. Because of this, there are potential trade-offs between the nutrition that cnidarian hosts gain from their symbionts and their ability to successfully defend themselves from pathogens. To investigate these potential trade-offs, we utilized the facultatively symbiotic polyps of the upside-down jellyfish *Cassiopea xamachana* and exposed aposymbiotic and symbiotic polyps to the pathogen *Serratia marcescens*. Symbiotic polyps had a lower probability of survival following *S. marcescens* exposure. Gene expression analyses 24 hours following pathogen exposure indicate that symbiotic animals mounted a more damaging immune response, with higher levels of inflammation and oxidative stress likely resulting in more severe disruptions to cellular homeostasis. Underlying this more damaging immune response may be differences in constitutive and pathogen-induced expression of immune transcription factors between aposymbiotic and symbiotic polyps rather than broadscale immune suppression during symbiosis. Our findings indicate that in facultatively symbiotic polyps, hosting symbionts limits *C. xamachana’*s ability to survive pathogen exposure, indicating a trade-off between symbiosis and immunity that has potential implications for coral disease research.

## 1. Introduction

Throughout the metazoan phylogeny, several taxa have evolved photosymbiosis or mutualistic relationships with photosynthetic organisms [1]. In these mutualisms, the symbionts provide their hosts with photosynthates, which often account for the bulk of the hosts’ nutrition, in exchange for nutrients such as nitrogen and the protection of being housed within the host [2,3]. Photosymbiosis is common throughout the cnidarian phylogeny, with the vast majority of symbiotic cnidarians forming an intracellular symbiosis with dinoflagellates in the family Symbiodiniaceae [2,4]. This symbiosis is best known for its vital role in coral reef ecosystems, as the nutrition provided by the symbionts allows the cnidarian hosts to live in oligotrophic environments that would otherwise be uninhabitable for them if they relied upon heterotrophy alone [5,6].

The extent to which the cnidarian host is reliant upon their symbionts for nutrition varies. This symbiosis can be obligate, as seen in tropical reef-building corals, facultative, as seen in some anemones and soft corals, or both depending on the life stage, as seen in the scyphozoan genus *Cassiopea* [5,7–9]. While all algal symbionts are housed intracellularly in a specialized acidic organelle called the symbiosome, the cell type in which the symbionts reside is variable [2,7,8]. This is likely owing to the complex evolutionary history of the cnidarian–algal symbiosis, which has independently evolved several times [4,9,10]. Members of the classes Hexacorallia and Octocorallia, which account for the vast majority of symbiotic cnidarians, house their symbionts in the gastrodermis [2,7]. This is in contrast to the more distantly related scyphozoans whose symbionts are housed in mobile cells called amoebocytes within the mesoglea [8].

The complete mechanisms of symbiosis establishment and maintenance within cnidarians are still unknown and may vary across the independently evolved symbioses [7,11]. Recognition of the algal symbionts likely occurs via pattern recognition receptors (PRRs), though many different classes of these receptors have been implicated [11]. However, there is evidence in soft and hard corals that lectins opsonize the symbionts prior to phagocytosis [7,12–14]. Following phagocytosis, non-compatible symbionts are expelled from the symbiont-hosting cells via vomocytosis, while compatible symbionts are retained to establish their intracellular niche [15]. The establishment of compatible symbionts is strongly associated with the suppression of the cnidarian hosts’ innate immune system [11,16–18]. Studies indicate that this immune suppression likely occurs via the suppression of the master immune regulator and transcription factor nuclear-factor kappa B (NFκB) or through the suppression of pathways upstream of NFκB [15,19,20]. This immune suppression persists in the symbiont-hosting cells in order to retain their symbionts [17–19].

Because symbiotic cnidarians suppress their immune systems while maintaining populations of intracellular symbionts, there is a potential trade-off between the nutrition these animals get from their symbionts and their ability to respond to pathogens. With recent increases in the frequency and severity of coral disease outbreaks, it is pertinent to understand how hosting symbionts influences cnidarians’ ability to defend themselves against pathogens [21–24]. Aposymbiotic *Exaiptasia diaphana* have been shown to be less susceptible to *Serratia marcescens* infection relative to their symbiotic counterparts [25]. This species has also shown marked differences in gene expression between symbiotic and aposymbiotic animals in response to *Vibrio coralliilyticus* [26]. However, it has not been established whether immune suppression and the subsequent trade-off between symbiosis and immunity are shared across independent evolutions of the cnidarian–algal symbiosis, given the differences in the symbiont housing cell type. Therefore, we tested the infection outcomes and responses of aposymbiotic and symbiotic *Cassiopea xamachana* to the known cnidarian pathogen *S. marcescens. C. xamachana* are benthic jellyfish that are facultatively symbiotic in their polyp life stage [27]. This facultative symbiosis can be leveraged to disentangle the role of symbiosis in pathogen-induced stress. We found that symbiotic *C. xamachana*, similar to *E. diaphana*, are more susceptible to bacterial infection relative to their aposymbiotic counterparts. To further investigate the mechanisms of this trade-off between symbiosis and immunity, we measured their acidic organelle activity and gene expression following pathogen exposure. These data give more insights into the trade-offs between symbiosis and immunity in cnidarians by identifying core shared responses and phenomena across the independently evolved symbioses.

## 2. Methods

### (a) Animal husbandry

*C. xamachana* polyps were obtained from the Dallas Children’s Aquarium and maintained at 27°C in 35 ppt artificial seawater (ASW). Polyps were fed *Artemia* nauplii and given water changes twice per week. Aposymbiotic polyps were generated by maintaining animals in the dark for a minimum of two months. Polyps were considered aposymbiotic when they had fewer than 10 symbionts visible via a dissecting microscope. To confirm that our aposymbiotic polyps had vastly reduced symbiont populations relative to symbiotic polyps, we imaged 10 polyps from each symbiotic state under an enhanced green fluorescent protein (eGFP) filter using a Zeiss imager Z2 microscope. The mean fluorescence of the bell of each polyp was quantified in ImageJ (v.1.53 t) by subtracting the mean background fluorescence from the mean total fluorescence. A *t*‐test was used to confirm a significant reduction in symbiont density (figure 1*a*; *p* = 2.12 × 10^−7^).

### (b) Survival analysis and quantification of acidic organelle activity

Polyps were fed *Artemia* nauplii 48 h prior to the start of all experiments. *S. marcescens* was cultured at 30°C for 24 h in a general ASW media [28]. The bacteria were pelleted, washed with 0.2 µm filtered ASW and diluted down to a concentration of 10^8^ colony-forming units (CFU)/ml prior to placing the polyps into the solution. Survival experiments had four treatment groups—symbiotic controls, aposymbiotic controls, symbiotic exposed and aposymbiotic exposed—and they were run at an ambient temperature of 27°C. Polyp mortality of each group was measured every 24 h following the start of the experiment. Mortality was defined as a polyp with the complete loss of bell structure. Differences in survival between treatment groups were tested using a Kaplan–Meier survival analysis using the R package survival and visualized with the R package survminer [29–31].

Acidic organelle activity was measured following 24 and 72 h of exposure to the respective treatments by incubating polyps in the dark in a solution of 5 µl ml^–1^ lysotracker red (Invitrogen L7528) in 0.2 µm filtered ASW for 30 min. Following the incubation period, animals were washed three times in 0.2 µm filtered ASW before being mounted onto slides and imaged at 5× under a red fluorescent protein (RFP) filter using a Zeiss imager Z2 microscope. Mean florescence of the bell of each polyp was quantified using Image J by subtracting the mean background fluorescence from the mean total fluorescence. A two-way ANOVA (mean fluorescence ~ symbiotic status × treatment) was used to test for differences in acidic organelle activity between treatment groups at 24 and 72 h.

### (c) RNA extractions and sequencing

Thirty non-clonal polyps per treatment group were exposed to their respective treatment conditions for 24 h. Polyps were then pooled in groups of five for a total of six replicates per treatment group, placed into 600 µl of 1× DNA/RNA shield (Zymo: R1100) and flash frozen in liquid nitrogen. Samples were homogenized and RNA was extracted using the Zymo RNA miniprep plus kit (R1057). Extracted RNA was sent to Novogene Co. Ltd. Following quality control, 18 samples proceeded to polyA tail capture and cDNA library preparation. The cDNA libraries were then sequenced on an Illumina NovaSeq 6000 using 150 bp paired-end sequencing.

### (d) Data analysis

Raw reads were trimmed using fastp (v.0.23.3) and mapped to the gene models of the *C. xamachana* draft genome using salmon (v.1.9.0) [32–34]. The corresponding proteome was annotated using both eggnogmapper (v.2.1.12) and STRING (taxon identifier STRG0A63JRD) [35,36]. Previously identified *C. xamachana* PRRs were identified in the proteome using blast [37,38]. To confirm that replicates hosted *C. xamachana’s* preferred symbiont genus, *Symbiodinium*, we measured the percentage of trimmed reads per sample mapping to the *C. xamachana* gene models as well as to transcriptomes representing *Symbiodinium, Breviolium, Cladocopium* and *Durusdinium* using BBsplit v.39.01 [34,39–43]. This method of determining dominant symbiont genus has been previously utilized in reef-building corals and was verified by ITS2 data [24].

tximport was used to format the reads and correct for biases in length and GC content [44]. Outlier analyses were performed through a principal component analysis (PCA) using PCAtools and a cluster dendrogram analysis from the wgcna R package, resulting in the exclusion of three samples from downstream analysis (electronic supplementary material, figure S1) [45,46]. Following the exclusion of outliers, a second PCA was performed [46]. The loadings for principal component 1 (PC1) were then used as a variable for gene ontology (GO) enrichment analysis using gomwu [47].

Differentially expressed genes (DEGs) were called using DESeq2 with the following model: ~symbiotic status + treatment + treatment × symbiotic status [48]. The appropriate contrasts in DESeq2 were used to separate DEGs from the following comparisons: symbiotic controls versus aposymbiotic controls, aposymbiotic exposed versus aposymbiotic controls, symbiotic exposed versus symbiotic controls and symbiotic exposed versus aposymbiotic exposed. The log fold change for each of these comparisons was then used for rank-based GO enrichment analysis using gomwu.

Signed gene co-expression networks were detected using weighted gene co-expression network analysis (wgcna) with a soft power of 9 and a minimum module size of 150 (electronic supplementary material, figure S2) [45]. Modules were then correlated to treatment and symbiotic status, while each contig was correlated to treatment. Of the modules significantly correlated to any given trait or combination of traits, the most significantly correlated modules to a given trait or traits were tested for functional enrichments. If no enrichments were found, the second most correlated module for a given trait was then tested for functional enrichments. Functional enrichments were tested using STRING against the background of all contigs with a mean expression >10 in the experiment [36]. Immune transcription factors were selected based on their significant differential expression in multiple gene expression comparisons and membership within modules most significantly correlated to a given trait or traits. A one-way ANOVA and subsequent Tukey honest significant difference (HSD) tests were run on the regularized log (rlog) normalized expression of these transcription factors to identify significant differences in expression between the four treatment groups.

## 3. Results

**Figure 1.**
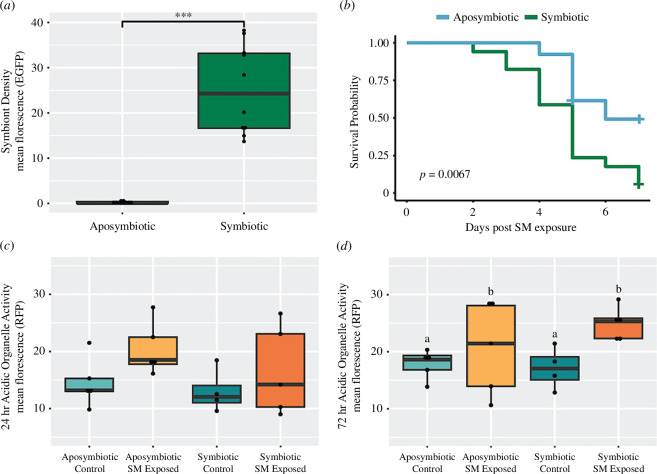
(*a*) Difference in symbiont density measured as mean eGFP florescence between symbiotic and aposymbiotic polyps. Points represent individual polyps’ mean eGFP florescence. *** *p*‐value < 0.001. (*b*) Kaplan–Meier survival plot comparing survival probability between symbiotic and aposymbiotic polyps following exposure to 10^8^ colony-forming units (CFU) of *S. marcescens*. Crosses indicate points where individuals lived past the end of *S. marcescens* (SM) exposure. (*c,d*) Boxplots of acidic organelle activity measured as mean RFP florescence at (*c*) 24 h and (*d*) 72 h following *S. marcescens* exposure. Letters denote significantly different groups with a *p*‐value < 0.05 resulting from a two-way ANOVA and Tukey *post hoc* test. Points represent individual polyps’ mean RFP florescence.

### (a) Survival analysis and acidic organelle activity

**Figure 2.**
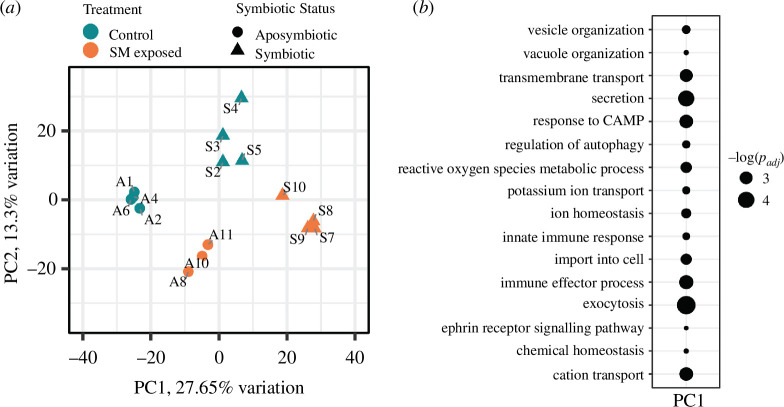
(*a*) PCA of rlog normalized gene expression. Teal points indicate control replicates, orange points indicate pathogen-exposed replicates, circular points indicate aposymbiotic replicates and triangular points indicate symbiotic replicates. SM, *S. marcescens.* (*b*) Rank-based gene ontology enrichments significantly positively associated with PC1. The size of each point is reflective of the −log adjusted *p*-value of its corresponding term.

Symbiotic polyps are significantly less likely to survive exposure to *S. marcescens* at a concentration of 10^8^ CFU ml^–1^ relative to their aposymbiotic counterparts (Kaplan–Meier survival analysis, *p* = 0.0067) (figure 1*b*). Only 5.9% of symbiotic polyps survived exposure to the pathogen, as compared to 53.8% of aposymbiotic polyps. No mortality was observed in the symbiotic control group or the aposymbiotic control group. Acidic organelle activity did not significantly vary between any of the treatment groups 24 h following *S. marcescens* exposure (figure 1*c*). However, it significantly increased 72 h following *S. marcescens* exposure (two-way ANOVA, *p* = 0.038), with no significant effect of symbiosis on this increase (figure 1*d*).

### (b) Gene expression analysis

**Figure 3.**
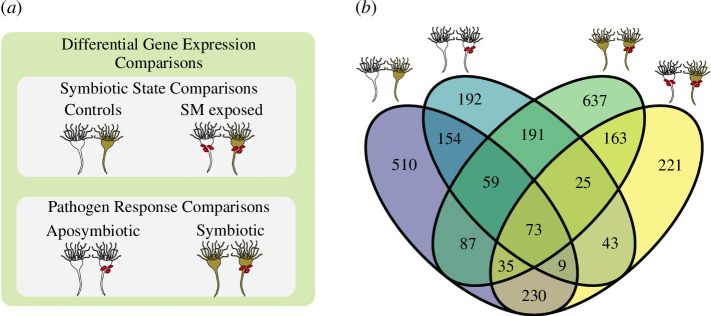
(*a*) Schematic of the four comparisons made during differential gene expression analysis separated into symbiotic state comparisons and pathogen response comparisons. SM, *S. marcescens.* (*b*) Venn diagram of overlapping differentially expressed genes between all differential gene expression comparisons. Specific comparisons indicated by polyp icons.

The Symbiodiniaceae transcriptome with the highest percentage of unambiguous mapped reads was *Symbiodinium* for all replicates. This indicates that all samples were dominated by *C. xamachana’s* preferred symbiont genus. An average of 11.8 million reads per replicate mapped to the *C. xamachana* gene models for an average mapping rate of 55.14% (electronic supplementary material S1). Removal of outliers resulted in 15 samples: four symbiotic control replicates, four symbiotic pathogen-exposed replicates, four aposymbiotic control replicates and three aposymbiotic pathogen-exposed replicates, for downstream analysis. After filtering genes with low expression in the dataset, the count data consisted of 16 989 expressed genes.

**Figure 4.**
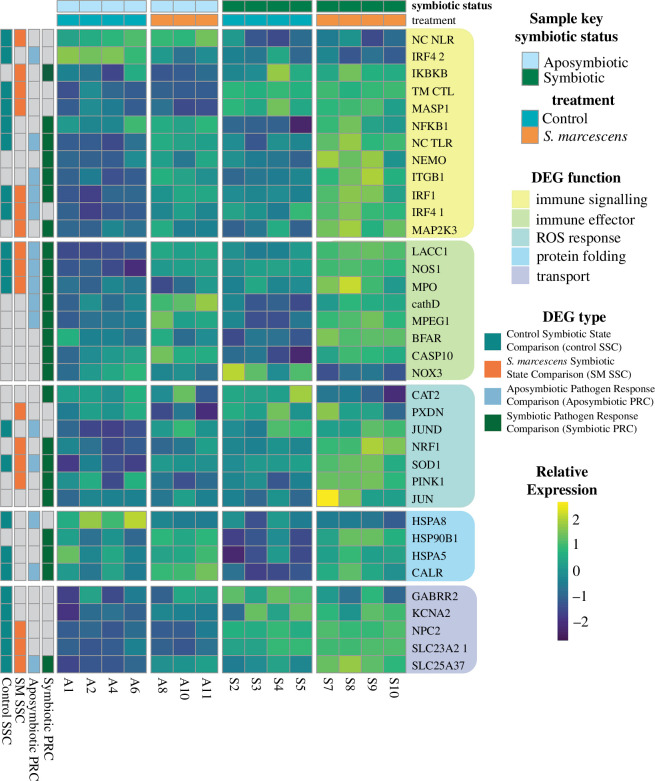
Heat map of select DEGs using relative rlog expression. Column key indicates symbiotic status/treatment of each section. Row key indicates which comparison(s) the gene is differentially expressed in. Rows are grouped by DEG function. abbreviations: NC NLR, non-canonical NLR; IRF4 2, IRF4 paralog 2, NC TLR- non-canonical TLR; IRF4 1, IRF4 paralog 1.

A PCA showed distinct grouping of the replicates by the treatment group across the first two principal components (PCs) (figure 2*a*). Notably, the symbiotic control replicates grouped most closely with aposymbiotic pathogen-exposed replicates across the first PC (PC1) (figure 2*a*). Thirty-two biological process GO terms were significantly (*p*_adj _< 0.01) positively associated with PC1, including processes related to ion homeostasis, reactive oxygen species (ROS) metabolism and innate immunity (electronic supplementary material, S2, figure 2*b*).

Four comparisons were made during differential gene expression analysis, falling into two categories: symbiotic state comparisons and pathogen response comparisons (figure 3*a*). Symbiotic state comparisons test for differences between the symbiotic states within the same treatment groups, comparing (i) aposymbiotic controls and symbiotic controls and (ii) aposymbiotic pathogen-exposed and symbiotic pathogen-exposed. Pathogen response comparisons test the response of each symbiotic state to *S. marcescens*, comparing (iii) aposymbiotic controls and aposymbiotic pathogen-exposed, and (iv) symbiotic controls and symbiotic pathogen-exposed. There were 1157 DEGs between aposymbiotic controls and symbiotic controls, 643 of which were annotated (electronic supplementary material, S3). Of these 1157 genes, 347 were differentially expressed in both symbiotic state comparisons, regardless of exposure to *S. marcescens* (figure 3*b*).

**Figure 5.**
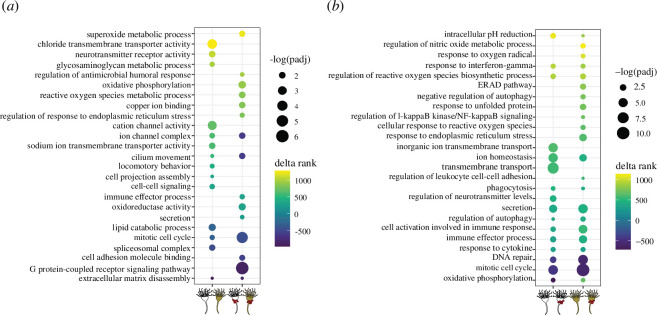
Curated set of rank-based gene ontology enrichments significantly differentially expressed in (*a*) symbiotic state comparisons and (*b*) pathogen response comparisons. Colour of each point indicates the delta rank of its corresponding term, while size indicates the –log-adjusted *p*‐value.

**Figure 6.**
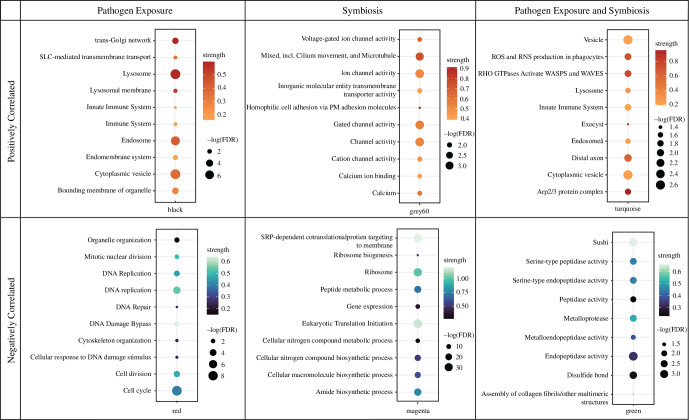
Curated set of STRING enrichments of WGCNA modules significantly correlated to symbiosis, pathogen exposure, or symbiosis and pathogen exposure. Colour of each point corresponds to the strength of the enrichment term, and the size of each point indicates the –log false discovery rate of the enrichment term.

**Figure 7.**
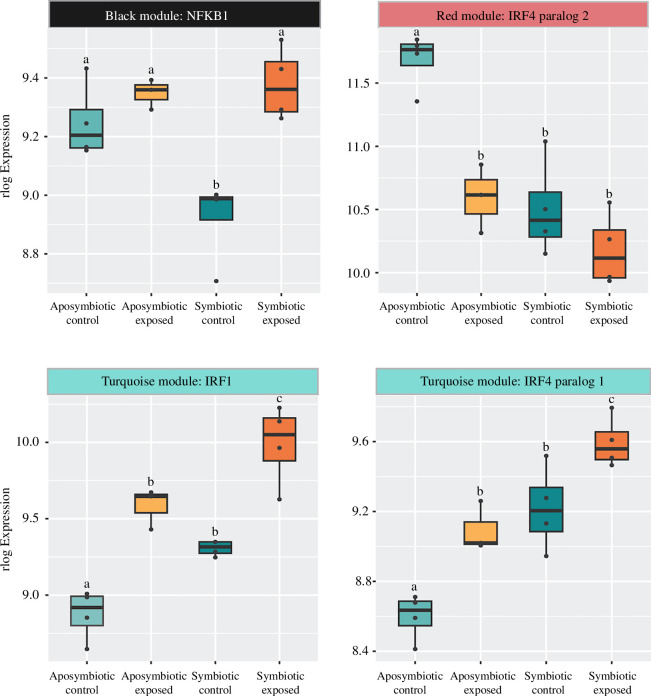
WGCNA module membership and box plots with the rlog expression of (*a*) NFκB, (*b*) IRF4 paralog 2, (*c*) IRF1 and (*d*) IRF4 paralog 1. Letters represent significantly different treatment groups with a *p*‐value < 0.05 resulting from a one-way ANOVA and Tukey post hoc test. Points represent individual replicates’ rlog expression.

Significant downregulation of NFκB occurred in symbiotic controls relative to aposymbiotic controls (figure 4). Several other genes associated with immune stress responses were significantly upregulated in symbiotic controls relative to their aposymbiotic counterparts, including nitric oxide synthase 1 (NOS1), interferon regulatory factor 1 (IRF1), interferon regulatory factor 4 paralog 1 (IRF4 paralog 1) and superoxide dismutase 1 (SOD1) (figure 4). Ranked GO enrichment analysis found 158 GO terms significantly upregulated and downregulated in the symbiotic controls relative to aposymbiotic controls (figure 5*a*, electronic supplementary material, S4). Several of these significantly upregulated enrichments in symbiotic controls are associated with ion transport and the nervous system (figure 5*a*). Additionally, lipid catabolism was downregulated in symbiotic controls relative to aposymbiotic controls (figure 5*a*).

When the aposymbiotic and symbiotic pathogen-exposed treatments were compared, there were 799 differentially expressed genes, 418 of which were annotated (electronic supplementary material, S3). IRF1, IRF4 *C*. *xamachana* paralog 1 (IRF4 paralog 1), nuclear respiratory factor 1 (NRF1) and SOD1 were all upregulated in symbiotic pathogen-exposed polyps relative to aposymbiotic polyps (figure 4). Rank-based GO enrichment analysis found evidence for a stronger immune response in symbiotic polyps relative to aposymbiotic polyps following pathogen challenge with the upregulation of GO terms indicative of immune effector secretion and oxidative stress (figure 5*a*, electronic supplementary material, S4). A total of 338 GO terms were significantly differentially expressed in this comparison (electronic supplementary material, S4).

The aposymbiotic polyps exposed to *S. marcescens* differentially expressed 746 genes relative to aposymbiotic controls, 442 of which were annotated (electronic supplementary material, S3). Many of these genes, such as IRF1, IRF4 paralog 1, SOD1 and macrophage expressed 1 (MPEG1), have functions in innate immunity (figure 4). Rank-based GO enrichment analysis found 414 differentially expressed GO terms between pathogen-exposed aposymbiotic polyps relative to aposymbiotic control polyps, including the upregulation of terms related to phagocytosis, inflammatory immune effectors and ROS biosynthesis (figure 5*b*, electronic supplementary material, S4).

Symbiotic polyps differentially expressed 1270 genes following pathogen exposure relative to symbiotic controls, 773 of which were annotated (electronic supplementary material, S3). There were 348 overlapping genes between the aposymbiotic pathogen response comparison and the symbiotic pathogen response comparison, including the upregulation of IRF1, SOD1 and MPEG1 (figure 4). Additionally, symbiotic polyps upregulated NFκB, catalase 2 (CAT2) and NRF1 in response to *S. marcescens* (figure 4). This comparison had 515 GO enrichments. Symbiotic polyps upregulated many of the same immune stress response terms as aposymbiotic polyps in response to *S. marcescens* (figure 5*b*, electronic supplementary material, S4). However, the pathogen response of symbiotic polyps also involved the upregulation of terms indicative of oxidative stress and endoplasmic reticulum (ER) stress (figure 5*b*).

Signed weighted gene co-expression network construction resulted in 23 modules of co-expressed genes. Of these 23 modules, seven had significant correlations to treatment, four had significant correlations to symbiotic status and two had significant correlations for treatment and symbiotic status (electronic supplementary material, figure S3). The module most negatively correlated to symbiosis (Green module, *r* = −0.9, *p* = 5 × 10^−6^) had STRING enrichments for terms related to assembly of collagen fibrils and peptidase activity (figure 6, electronic supplementary material, S5). The module with the highest positive correlation to symbiosis (Brown module, *r* = 0.92, *p* = 8 × 10^−7^) did not have any significant STRING enrichments; however, the module with the next highest positive correlation to symbiosis (Grey60 module, *r* = 0.59, *p* = 0.02) was enriched for several terms related to ion transport (figure 6, electronic supplementary material, S5). The module most negatively correlated to *S. marcescens* exposure (Magenta module, *r* = −0.81, *p* = 3 × 10^−4^) was enriched for terms related to nitrogen metabolism. In contrast, the module most positively correlated to *S. marcescens* exposure (Black module, *r* = 0.93, *p* = 7 × 10^−7^) contained the transcription factor NFκB (module membership (mm) = 0.74, *p* = 0.002) and was enriched for the innate immune system and the endomembrane system (figures 6 and 7*a*). The red module was negatively correlated to both symbiosis (*r* = −0.81, *p* = 3 × 10^−4^) and *S. marcescens* exposure (*r* = −0.63, *p* = 0.01). This module contains IRF4 paralog 2 (mm = 0.85, *p* = 6.01 × 10^−5^) and was enriched for the cell cycle and cytoskeleton organization (figures 6 and 7*b*). Finally, the turquoise module was positively correlated to both symbiosis (*r* = 0.67, *p* = 0.007) and pathogen exposure (*r* = 0.76, *p* = 0.001). This module contained two immune transcription factors, IRF1 (mm = 0.94, *p* = 2.93 × 10^−7^) and IRF4 paralog 1 (mm = 0.87, *p* = 2.22 × 10^−5^), and was enriched for the innate immune system and oxidant production (figures 6 and 7*c,d*, electronic supplementary material, S5).

## 4. Discussion

The lower survival of symbiotic polyps following *S. marcescens* exposure indicates the presence of a trade-off between the nutritional benefit of hosting symbionts and immunocompetence in facultatively symbiotic *C. xamachana*. We found that hosting Symbiodiniaceae alters both constitutive and pathogen-induced *C. xamachana* gene expression. Symbiosis in *C. xamachana* altered the constitutive expression of metabolism, ion transport and innate immunity. When exposed to *S. marcescens*, symbiotic *C. xamachana* upregulated a stronger ROS and immune effector response, likely disrupting protein homeostasis in the endomembrane system and leading to low survival rates. We hypothesize that differences in constitutive and pathogen-induced expression of immune transcription factors drive symbiotic polyps’ greater susceptibility to bacterial pathogens.

In comparing the two control groups of our study, we found several notable differences in the constitutive expression of symbiotic and aposymbiotic *C. xamachana*. Several genes involved in transport and metabolism were differentially expressed in symbiotic animals relative to their aposymbiotic counterparts. Consistent with findings in the symbiotic sea anemone *E. diaphana*, our symbiotic controls downregulated lipid catabolism relative to their aposymbiotic counterparts. Previous studies attribute these expression changes in lipid metabolism to be indicative of increased energy stores and thus characteristic of stable symbiosis [49,50]. Interestingly, we found no evidence of constitutive shifts in symbiotic polyps’ expression of nitrogen metabolism in any of our analyses. This is notable because shifts in nitrogen metabolism, specifically ammonium recycling via the glutamine synthetase/glutamate synthase system, are a well-established occurrence in symbiotic anthozoans and thought to be a mechanism of symbiont population control [9,12,17,51–54]. Isotopic studies have found that while *C. xamachana’*s symbionts have limited access to nitrate *in hospite*, they retain access to ammonium derived from heterotrophic feeding [55,56]. Our results support this finding, as GLUL, a gene responsible for the removal of ammonium by converting it to glutamine, is constitutively downregulated in symbiotic polyps. This same gene is upregulated in symbiotic *E. diaphana* and overexpressed in the symbiont-hosting cells of *Stylophora pistillata* and *Xenia* sp. [9,12,51,57]. The lack of transcriptomic signatures of constitutive changes to nitrogen metabolism in symbiotic *C. xamachana* polyps could be owing to their different symbiont-hosting cell type and their potential use of their bacterial microbiome to limit symbiont access to dissolved inorganic nitrogen [55,56].

We found extensive evidence for the constitutive differential regulation of ion transport while in a symbiotic state, with both the ranked GO enrichment analysis between control groups and the WGCNA module positively correlated to symbiosis having enrichments involving the nervous system and transmembrane ion transport. Similar expression patterns have been observed in *E. diaphana*, with many of the upregulated genes associated with ion transport (KCNA2, GAABRR2, CNTNAP4 in our study) functioning to decrease membrane excitability [51,58–60]. Several intracellular protozoan parasites cause similar changes to their host’s ion transport, often to prevent the host cell from producing nitric oxide [61–63]. Symbiodiniaceae may employ similar strategies while residing intracellularly, as high levels of host-derived nitric oxide have been shown to lead to symbiosis breakdown [64,65]. However, nitric oxide synthase is upregulated in symbiotic controls relative to aposymbiotic controls, so the changes in expression of these ion transporters may serve a different function in the maintenance of symbionts.

Our data do not suggest that symbiotic animals have large-scale downregulation of immunity, as there were no GO terms related to immunity that were significantly differentially expressed between the two control groups. This, along with the PCA showing symbiotic controls grouping with the aposymbiotic pathogen-exposed replicates across PC1, which is enriched for genes with innate immune response GO annotations, indicates that broadscale immune suppression owing to hosting symbionts is unlikely to be driving differences in survival rates between symbiotic states as has been hypothesized in other symbiotic cnidarians [11,16]. However, we did find several immune transcription factors with significantly different constitutive expression between the symbiotic states. Two of these transcription factors, NFκB and IRF4 paralog 2, have significantly lower expression in symbiotic controls relative to aposymbiotic controls. This NFκB expression pattern has been found in symbiotic anemones [19,20]. Additionally, there is evidence that stony corals negatively regulate NFκB pathways while hosting symbionts, indicating that NFκB downregulation is a common characteristic of the cnidarian–algal symbiosis across independent evolutions of the trait [17,18].

The other two immune transcription factors differentially regulated between the control groups are IRF1 and IRF4 paralog 1, which have significantly higher expression in symbiotic controls relative to aposymbiotic controls. Interferon regulatory factors have yet to be implicated in cnidarian symbiosis, but similar to NFκB these immune transcription factors are capable of mounting pro-inflammatory immune responses [66,67]. IRF expression can be both constitutive, providing basal levels of defence against microbes, and inducible, following danger-associated molecular pattern recognition [67–69]. Our data indicate that *S. marcescens* exposure can induce IRF1 and IRF4 paralog 1 expression, but not IRF4 paralog 2. As such, aposymbiotic animals are only constitutively upregulating a single immune transcription factor with *S. marcescens*-induced expression (NFκB), whereas symbiotic animals are constitutively upregulating two (IRF1 and IRF4 paralog 1). The differential regulation of these transcription factors may underlie why symbiotic polyps are less likely to survive *S. marcescens* exposure.

The pathogen response of symbiotic *C. xamachana* shared some similarities with aposymbiotic *C. xamachana*. Regardless of symbiotic state, *C. xamachana* polyps upregulated GO terms indicative of the secretion of immune effector proteins. One such immune effector was MPEG1, a bactericidal protein [70,71]. However, many of these same GO terms are significantly upregulated in symbiotic pathogen-exposed polyps relative to aposymbiotic pathogen-exposed polyps, indicating that symbiotic animals are likely mounting a stronger immune effector response than their aposymbiotic counterparts. This could be owing to the utilization of different pro-inflammatory immune pathways in the aposymbiotic and symbiotic pathogen responses. Both aposymbiotic and symbiotic polyps are upregulating ‘response to interferon gamma’ when exposed to *S. marcescens*. As cnidarians lack interferons, this GO enrichment likely suggests the upregulation of IRFs and other genes under transcriptional control of IRFs [66,72]. This is supported by both symbiotic states upregulating IRF1 in response to the pathogen. However, as symbiotic polyps have higher baseline expression of IRF1 and IRF4 paralog 1, symbiotic pathogen-exposed polyps have significantly higher expression of these transcription factors relative to aposymbiotic pathogen-exposed polyps. This is further supported by both these transcription factors belonging to the turquoise module, which is significantly positively correlated to symbiosis and *S. marcescens* exposure. Additionally, symbiotic polyps are upregulating the GO term ‘regulation of IκBK/NFκB signaling’ in response to *S. marcescens* as well as NFκB and one of its activators, NFκB essential modulator (NEMO). However, symbiotic polyps are also upregulating IκBKB, the inhibitor of NFκB, in response to *S. marcescens*. As NFκB is often a component of the initial immune signalling following the introduction of a stressor, this could suggest that at 24 h following *S. marcescens* exposure, symbiotic polyps are transitioning to different immune stress response pathways [73,74].

Stronger pro-inflammatory immune signalling in symbiotic polyps relative to aposymbiotic polyps could explain why gene expression signatures of oxidative stress are higher in pathogen-exposed symbiotic polyps relative to their aposymbiotic counterparts [75]. While the pathogen response of both symbiotic states included the upregulation of the GO term ‘regulation of ROS biosynthetic process’, only the symbiotic pathogen response included the upregulation of GO terms associated with responding to ROS. Additionally, the turquoise module, which is positively correlated to both pathogen exposure and symbiosis, is enriched for oxidant production. Higher levels of oxidants in pathogen-exposed symbiotic polyps are further supported by their significantly higher expression of the antioxidant SOD1 and the oxidative stress transcription factor NRF1 relative to pathogen-exposed aposymbiotic polyps [76,77]. Pro-inflammatory factors can induce ROS production directly and indirectly [78,79]. Higher demands for immune effector secretion can result in the upregulation of oxidative phosphorylation during immune responses [80]. The upregulation of oxidative phosphorylation in symbiotic pathogen-exposed polyps relative to their aposymbiotic counterparts may be another contributor to the higher transcriptional signatures of oxidative stress in the symbiotic pathogen response. Together, the stronger upregulation of immune effector responses, oxidative stress responses and oxidative phosphorylation in symbiotic polyps indicates that their immune response to *S. marcescens* likely results in more severe disruptions to cellular homeostasis relative to aposymbiotic polyps [78–80].

Our gene expression data indicate that symbiotic polyps experience disruptions to endomembrane system homeostasis and the protein folding environment within the cell. Oxidative stress, high secretory demand and immune effector proteins are all capable of disrupting the protein folding environment within the ER and are more highly upregulated in symbiotic pathogen-exposed replicates relative to aposymbiotic pathogen-exposed replicates [81–83]. Symbiotic polyps upregulated both the endoplasmic-reticulum-associated protein degradation (ERAD) pathway and an unfolded protein response (UPR). These pathways are both indicative of disruptions to protein homeostasis and ER stress [84]. The ERAD pathway is responsible for removing misfolded or unfolded proteins from the ER. If these misfolded and/or unfolded proteins accumulate, they will trigger the UPR [84,85]. In response to the accumulation of misfolded and/or unfolded proteins, the UPR reduces protein synthesis and upregulates chaperonins to attempt to refold or repair the misfolded proteins [81,84]. Any proteins unable to be folded are degraded via either autophagy or the ERAD pathway [81,82,84]. If the cell is unable to correctly repair or refold its proteins or the misfolded proteins accumulate and are not able to be degraded, the UPR transitions into a cell death pathway [82,86]. Given that symbiotic polyps have considerably more disruptions to protein homeostasis at 24 h following *S. marcescens* exposure, they likely are transitioning to cell death pathways sooner than aposymbiotic polyps, resulting in lower survival.

Differential expression of genes associated with autophagy is a common component of cnidarian immune responses, particularly at relatively early timepoints following pathogen exposure or in disease-resistant coral species that are not as severely impacted by a given pathogen [87,88]. In the context of innate immunity, autophagy is cytoprotective and anti-inflammatory, counteracting the damage that secreted inflammatory factors can cause to mitochondria and the endomembrane system [89]. Both symbiotic and aposymbiotic polyps significantly changed their regulation of autophagy and had increased acidic organelle activity following exposure to *S. marcescens*. However, given the signatures of higher oxidative stress and ER stress in symbiotic polyps, it is likely that upregulation of autophagy is insufficient to counteract the damage caused by their immune response, resulting in the negative regulation of autophagy and transition to cell death pathways [86,89].

## 5. Conclusions

Together, our data demonstrate that there is a trade-off between photosymbiosis and immunocompetence in the facultatively symbiotic polyps of *C. xamachana*. Underlying this trade-off may be the differential regulation of immune transcription factors both constitutively and in response to *S. marcescens* exposure rather than broadscale immune suppression in symbiotic animals. Symbiotic *C. xamachana* mount a stronger and more damaging immune response following pathogen exposure, resulting in higher levels of oxidative stress, greater disruptions to cellular homeostasis and ultimately decreased survival rates. The trade-off between symbiosis and immunity seems to be shared across independent evolutions of facultative cnidarian–algal symbiosis, as *E. diaphana* are also more susceptible to *S. marcescens* in a symbiotic state [25]. There likely are more complexities to the nutritional aspect of this trade-off, as starvation has been shown to influence cnidarian immune gene expression [25,90]. With the expanding threat of disease to coral reef ecosystems, this trade-off could be a major factor in coral disease susceptibility and dysbiosis via bleaching or nutrient pollution. The cost of hosting symbionts should be investigated further and potentially incorporated into the paradigms of coral disease research [21,91].

## Data Availability

Microscopy images, survival data, all shell scripts, and all R code used are available in the Data Dryad Repository [92]. All raw sequence data and associated sample metadata are available on the NCBI SRA database (BioProject ID PRJNA1077944). Publicly available data used in this study include the draft *C. xamachana* genome gene models (https://mycocosm.jgi.doe.gov/Casxa1/Casxa1.home.html). Supplementary material is available online [93].
